# NT‐4/5 antagonizes the BDNF modulation of corticostriatal transmission: Role of the TrkB.T1 receptor

**DOI:** 10.1111/cns.13091

**Published:** 2019-01-21

**Authors:** Francisco M. Torres‐Cruz, Israel César Vivar‐Cortés, Isaac Moran, Ernesto Mendoza, Victor Gómez‐Pineda, Francisco García‐Sierra, Elizabeth Hernández‐Echeagaray

**Affiliations:** ^1^ Laboratorio de Neurofisiología del Desarrollo y la Neurodegeneración UBIMED FES‐Iztacala Universidad Nacional Autónoma de México Ciudad de México México; ^2^ Departamento de Biología Celular CINVESTAV Mexico City Mexico

## Abstract

Neurotrophins are related to survival, growth, differentiation and neurotrophic maintenance as well as modulation of synaptic transmission in different regions of the nervous system. BDNF effects have been studied in the striatum due to the trophic role of BDNF in medium spiny neurons; however, less is known about the effects of NT‐4/5, which is also present in the striatum and activates the TrkB receptor along with BDNF. If both neurotrophins are present in the striatum, the following question arises: What role do they play in striatal physiology? Thus, the aim of this study was to determine the physiological effect of the sequential application and coexistence of BDNF and NT‐4/5 on the modulation of corticostriatal synapses. Our data demonstrated that neurotrophins exhibit differential effects depending on exposure order. BDNF did not modify NT‐4/5 effect; however, NT‐4/5 inhibited the effects of BDNF. Experiments carried out in COS‐7 cells to understand the mechanisms of this antagonism, indicated that NT‐4/5 exerts its inhibitory effect on BDNF by upregulating the TrkB.T1 and downregulating the TrkB‐FL isoforms of the TrkB receptor.

## 1 INTRODUCTION

Neurotrophins are growth factors with high homology in their sequence and structure. Neurotrophins are involved in neural activities, such as synapse formation, synapse modulation and neuronal plasticity.^1^, ^2^ Neurotrophin actions depend on the activation of the tropomyosin receptor kinase (Trk) family and the low‐affinity p75 receptor.^3^ The neurotrophins brain‐derived neurotrophic factor (BDNF) and neurotrophin‐4/5 (NT‐4/5) participate in synapse efficacy, and their final effects depend on the neuronal type, temporal timing of the physiological response and maturity of the experimental organism.^4^, ^5^, ^6^, ^7^ In the neuromuscular plate, these neurotrophins potentiate synaptic activity^8^, ^9^, ^10^; additionally, they modulate excitatory synaptic transmission in the nervous system through the activation of TrkB receptors.^9^, ^11^, ^12^, ^13^ TrkB receptors in turn trigger the MAPK, PI3K, and PLC‐γ signaling pathways in glutamatergic synapses.^14^, ^15^, ^16^, ^17^

In the striatum, BDNF, NT‐4/5, and TrkB receptors are present.^18^ The striatum receives trophic support from BDNF, which is synthesized in the cortex and transported to the striatum through the corticostriatal pathway.^19^ Less is known about the cells that synthesize NT‐4/5, but BDNF and NT‐4/5 are differentially expressed throughout striatal postnatal development. While BDNF expression decreases, NT‐4/5 increases in the first postnatal month in mice,^20^ suggesting that NT‐4/5 may play a major physiological role during adulthood. We have previously shown that BDNF and NT‐4/5 independently potentiate corticostriatal transmission to different degrees,^21^ but it is unknown how both neurotrophins affect transmission when they are coexpressed. What is the purpose of the coexistence of two neurotrophins that activate the same Trk receptor in the same nucleus? Would TrkB sequential stimulation result in the same physiological response? This study evaluates the physiological implications for corticostriatal synaptic transmission if either BDNF → NT‐4/5 or NT‐4/5 → BDNF responses take place. As neurotrophin effects depend on signaling pathway activation promoted by TrkB stimulation, MAPK, PI3K, and PLC‐γ signaling was evaluated. Furthermore, TrkB stimulation may activate at least four isoforms: a catalytic full‐length form of TrkB (TrkB‐FL) and three truncated isoforms lacking the kinase domain, including TrkB.T1, TrkB.T2, and TrkB.T‐Shc.^12^ Then, the expression levels of TrkB‐FL, TrkB.T1, and p‐TrkB in striatal tissue and a cell system were evaluated after neurotrophin treatment. Our experiments show, for the first time, that NT‐4/5 inhibits the effects of BDNF by modifying the expression levels of the TrkB.T1 and TrkB‐FL isoforms.

## 2 METHODS

Male C57BL/6 mice (ENVIGO, México) 35 days old at the beginning of the experiments were used. The mice were housed in groups of five in Plexiglas boxes at room temperature (24‐26°C) under a 12:12 hours light/dark cycle with free access to food and water. The experimental procedures followed the national and international regulations for the care and use of experimental animals and were approved by the local bioethic**s** committee.

### 2.1 Reagents

BDNF and NT‐4/5 (PreProtech Inc, Rocky Hill, NJ, USA) were used in a concentration of 50 ng/mL (neurotrophins were reconstituted in water 1.0 mg/mL and diluted in phosphates Buffer, 0.1 mol/L, pH 7.4, following vendor instructions), and the rest of reagents were purchased from SIGMA‐Aldrich Co. LLC (St Louis, MO) unless otherwise stated.

### 2.2 Preparation of striatal slices for electrophysiological recordings

The mice were anesthetized with halothane; then, they were decapitated, and their brains removed and placed in ice‐cold (4°C) and oxygenated (95% O_2_, 5% CO_2_) low calcium saline solution (in mmol/L; 130 NaCl, 3 KCl, 1 CaCl_2_, 5 MgCl_2_, 1.25 NaH_2_PO_4_, 26 NaHCO_3_, and 10 glucose; 298‐300 mOsm, pH 7.4). Sagittal brain slices (400 µm) containing the striatum, were obtained in the low calcium saline with a vibroslicer (Ted Pella Inc, Redding, CA, USA). The slices were maintained at room temperature (25°C) in oxygenated physiological saline for 1 hour prior to recording.

### 2.3 Electrophysiological recordings

The electrophysiological experiments were performed in a recording chamber perfused with physiological saline (in mmol/L; 125 NaCl, 3 KCl, 2 CaCl_2_, 1 MgCl_2_, 26 NaHCO_3_, 0.2 thiourea, 0.2 ascorbic acid and 10 glucose, pH 7.4), constantly bubbled (95% O2, 5% CO2). (−)‐Bicuculline methiodide (10 µmol/L) was used in all experiments to block GABAergic inhibitory responses. A stimulus (5‐30 V, 50‐200 μs) generated by an isolated voltage stimulator (Digitimer DS2, Letchworth Garden City, UK) was administered through a tungsten bipolar electrode (CBCEE75, FHC Inc, Bowdoinham ME, USA) placed in the corpus callosum. The amplitude and duration of the stimulus were held at 45%‐50% of the maximum amplitude response of the population spike. The population spikes were recorded in the dorsal striatum at 32‐34°C using borosilicate glass micropipettes (30‐30‐1; FHC Inc) filled with isotonic solution (NaCl, 0.9%). The signal was amplified (AC GRASS P15, Quincy, MA, USA) and filtered (1 kHz) with an interface coupled to a PC running program in Lab View 6.1 (National Instruments, Austin, TX, USA).

### 2.4 Stimulation protocol

A paired‐pulse protocol was used to analyze the pre‐ or postsynaptic mechanisms of the neurotrophic effect. Then, a pair of stimuli (S1, S2) with the same duration and intensity was administered through the bipolar electrode with 30‐50 ms intervals. The population spikes were recorded for 10 minutes before neurotrophin treatment. Data were digitized, stored and analyzed offline with the aid of Microcal Origin, 9.1 (Microcal Origin Lab Corp., Northampton, MA, USA).

### 2.5 Western blot analysis

To evaluate TrkB receptors and PLC signaling, cerebral slices containing the striatum were incubated at RT and bubbled (95% O_2_‐5% CO_2_) in the physiological saline in the presence of Bicuculline; then, the slices were exposed to (a) BDNF (50 ng/mL), (b) BDNF (50 ng/mL) → NT‐4/5 (50 ng/mL), (c) NT‐4/5 (50 ng/mL), or (d) NT‐4/5 (50 ng/mL) → BDNF (50 ng/mL) for 10 or 30 minutes. Then, the striatum was obtained and manually homogenized in lysis buffer (TRIS‐HCl 26 mmol/L, 1% Triton x‐100, glycerol 1.3 mol/L, NaCl 130 mmol/L) and phosphatase inhibitor complete mini tabs (Roche, Indianapolis, IN, USA). The homogenate samples were collected, centrifuged (5 minutes × 2812.5 *g*), and the supernatant was stored at −70°C. A total of 10‐30 µg of protein quantified by the Bradford method was loaded onto 10% polyacrylamide gels for electrophoresis. The proteins were transferred to polyvinylidene fluoride (PVDF) membranes and incubated with primary antibodies against phosphor‐Tyr816‐TrkB (1:1000), TrkB (1:1000), phospho‐Tyr783‐PLC‐γ1 (1:1000), PLC‐γ1 (1:1000), phospho‐Tyr759‐PLC‐γ2 (1:1000), PLC‐γ2 (1:1000), and actin (1:1000) for 12 hours at 4°C. The membranes were then incubated with hydrogen peroxidase‐conjugated (HRP) secondary antibodies for 1‐2 hours at room temperature. All primary antibodies were purchased from Cell Signaling (Technology, Danvers, MA, USA), and peroxidase‐goat antimouse IgG and peroxidase‐goat antirabbit IgG secondary antibodies were purchased from Invitrogen (Camarillo, CA, USA) and ThermoFisher Scientific (Rockford, IL, USA), respectively. Protein detection was performed via the chemiluminescence method (Gel Documentation System, Bio Sens SC 645; Shanghai Bio‐Tech Co., Ltd, Shanghai, China), and images were analyzed with ImageJ software (NIH) for densitometry.

### 2.6 Cell culture and transfection

The COS‐7 cell line obtained from the ATCC (Manassas, VA, USA) was grown in DMEM; glucose (1/1) supplemented with 10% fetal bovine serum (FBS; GIBCO, Grand Island, NY, USA); 2 mmol/L L‐glutamine; 100 U/mL penicillin; and 100 μg/mL streptomycin, and the cells were maintained under a humidified atmosphere (5% CO_2_, 37**°**C). When the cells reached 50%‐60% confluence, the medium was changed to FBS‐free Optimem specialized medium (GIBCO), and the cells were transiently cotransfected with 1 μg of DNA from the TrkB constructs (plasmid pGFP‐N1‐TrkB [Addgene #32500] and pRc/CMV HA‐TrkB.T1 [Addgene #39980]) by using the Lipofectamine® 2000 reagent, following the manufacturer's instructions (Invitrogen). After transfection (4 hours), the cells were postincubated in fresh 10% FBS supplemented with DMEM medium and kept at 37**°**C for 48 hours. Under this transfection protocol, the efficiency was 40%. Thereafter, transfected cells were washed in ice‐cold phosphate‐buffered saline (PBS, pH 7.4) and processed for Western blotting.

### 2.7 Analysis of cells extracts by electrophoresis and Western blotting

After cell transfection and neurotrophin treatment, cell cultures were washed twice with PBS, scraped, lysed in radioimmunoprecipitation assay (RIPA) buffer containing a cocktail of protease inhibitors (in mmol/L: 150 NaCl, 50 TRIS, pH 8.0, 1 PMSF, 100 NaF, 1 Na_3_VO_4_; 1% Triton X‐100, 0.5% sodium deoxycholate, and 2 μg/mL complete; Roche, Indianapolis, IN, USA) and centrifuged (12 000 *g* × 10 minutes). The supernatant was collected, and protein content determined by the mini‐Bradford assay (Bio‐Rad Laboratories Inc, Hercules, CA, USA). A total of 30 μg of protein was mixed in 5× sample buffer (TRIS‐HCL 250 mmol/L pH 6.8, sodium dodecyl sulfate (SDS) 10%, bromophenol blue 0.5%, β‐mercaptoethanol 12.5%, and glycerol 50%) and boiled (95°C, 5 minutes). Proteins were separated by electrophoresis on a 10% SDS‐polyacrylamide gel (SDS‐PAGE) and transferred onto a nitrocellulose membrane for immunoblotting analysis. Membranes were blocked in 10% nonfat dried milk in PBS or TBS‐0.1% Tween 20 (PBS‐tw or TBS‐tw) overnight at 4°C and incubated for 12 hours in primary antibodies diluted in PBS‐tw or TBS‐tw. After washing, incubation with the corresponding HRP‐conjugated secondary antibodies to either mouse or rabbit was carried out for 1‐2 hours (RT). Bands of immunoreactive proteins were visualized as described above.

### 2.8 Data analysis

Data were analyzed with Sigma Plot 12.3 (Systat Software, Inc, San Jose, CA, USA) using parametric or nonparametric one‐way ANOVA, Data followed by post hoc analysis. Significance was set at *P* < 0.05, and the results are expressed as the mean ±SEM, unless otherwise specified.

## 3 RESULTS

### 3.1 NT‐4/5 blocks BDNF synaptic augmentation of corticostriatal synaptic transmission

**Figure 1:**
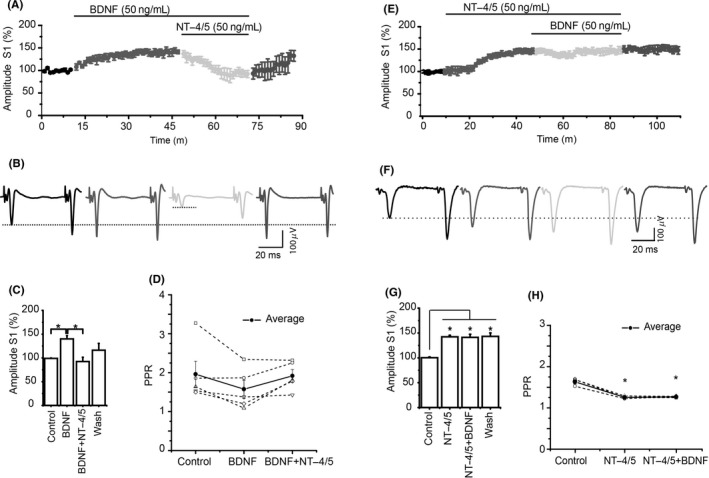
Modulation of synaptic transmission by BDNF and NT‐4/5. A, Temporal course of the population spike amplitude average in BDNF → NT‐4/5 experiments. BDNF increased the spike amplitude compared to control, but NT‐4/5 reverse the spike amplitude to control levels. The population spike recovers its amplitude after washing the neurotrophins. Lines indicate, time and concentration of BDNF and NT‐4/5. B, Representative traces of population spikes in control (Bicuculline 10 µmol/L), BDNF, BDNF → NT‐4/5, and wash. C, Data average of the synaptic spike amplitude from the last 10 minutes in each experimental condition is displayed in the bars (*F*_3,19_ = 5.485, *P* = 0.009, ANOVA with a post hoc Holm Sidak‐method). D, PPR analysis did not show differences among groups. Dashed lines are the individual values of PPR of each experiment and solid line illustrates average values. E, Temporal course of the population spike amplitude average in control, NT‐4/5 → BDNF experiments. NT‐4/5 increased the amplitude of population spike compared to control. The addition of BDNF did not change the amplitude increase produced by NT‐4/5. F, Representative traces of population spikes in control, NT‐4/5, NT‐4/5 → BDNF, and wash. G, Average of the spike amplitude from each condition is displayed in the bars (*F*_3,11_ = 17.766, *P* < 0.001, ANOVA with a post hoc Holm Sidak‐method). H, PPR Analysis exhibited differences in NT‐4/5 and NT‐4/5 → BDNF compared to control (*F*_3,11_ = 45.690, *P* < 0.00, ANOVA with a post hoc Holm Sidak‐method) *P < 0.05 (n = 5; Mean ± SE)

With a stable recording, either BDNF or NT‐4/5 was applied to the recording bath. Figure 1A and B shows that BDNF increased spike amplitude in response to S1 compared to the control, as we previously reported**.** 21 However, when NT‐4/5 was administered in the presence of BDNF, the spike amplitude significantly decreased (Figure 1C). This effect implies that NT‐4/5 antagonizes the effect of BDNF on corticostriatal transmission. PPR analysis (S2/S1) did not show significant differences, suggesting that both neurotrophins modulate corticostriatal transmission via postsynaptic mechanisms (Figure 1D).

### 3.2 NT‐4/5 modulation on corticostriatal transmission was not modified by BDNF

Next, we sought to evaluate whether BDNF was able to antagonize the effect of NT‐4/5 on corticostriatal transmission. The application of NT‐4/5 to the bath increased the spike amplitude compared to the spike amplitude in control condition. The addition of BDNF to the bath did not change the modulatory effect of NT‐4/5, suggesting that once NT‐4/5 activated the TrkB receptor, BNDF did not have any further modulatory effect (Figure 1E‐F). NT‐4/5 or NT‐4/5 → BDNF administration significantly changed PPR compared to the control PPR, suggesting a presynaptic mechanism (Figure 1H).

### 3.3 Signaling pathways involved in the neuromodulation of BDNF in corticostriatal synapses

**Figure 2:**
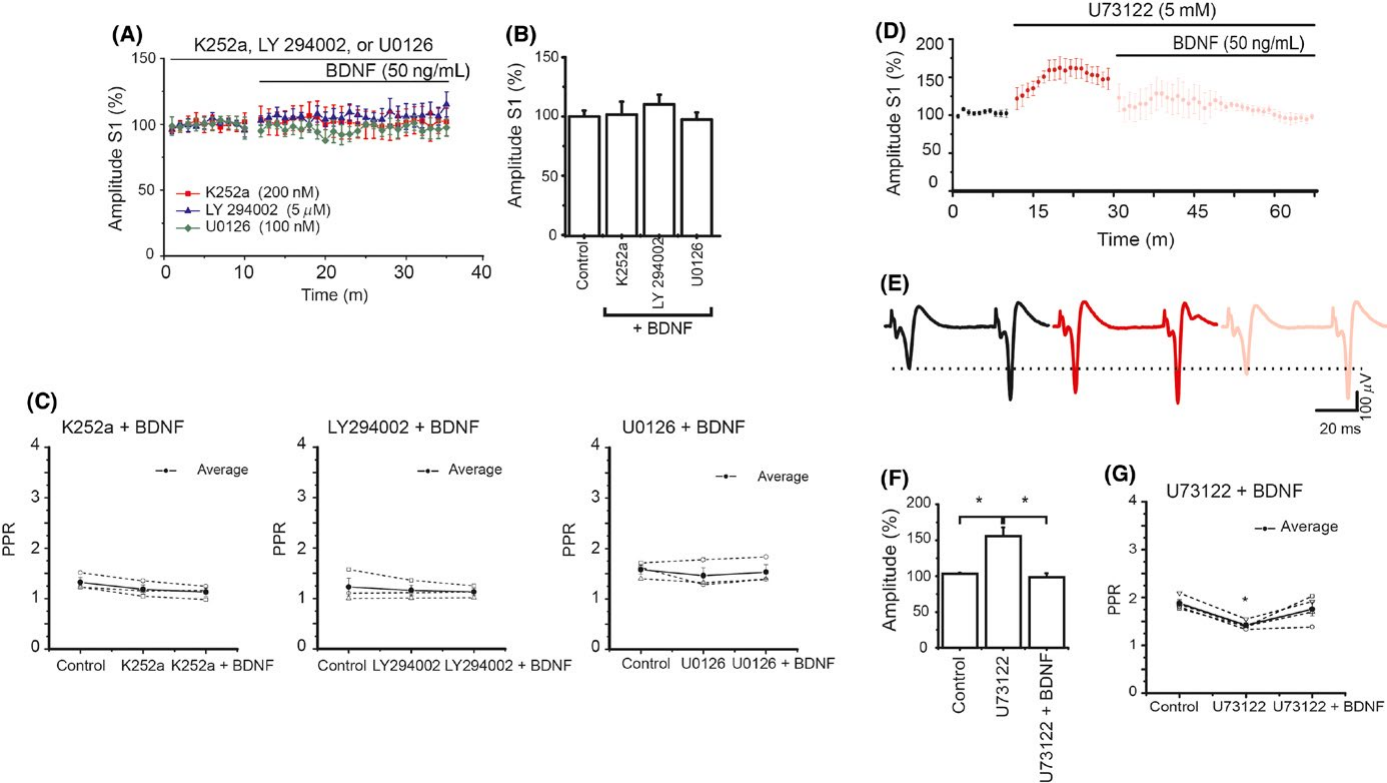
Signaling pathways associated with BDNF effects. A, Summary of the time course of the population spike amplitude in the presence of the inhibitors of Trk receptor, PI3K, and MAPK K252a (200 nmol/L), LY294002 (5 µmol/L), and U0126 (100 nmol/L), all dissolved in 0.05%‐0.1% DMSO were followed by BDNF. Each experimental condition was normalized to the inhibitor effect. These inhibitors prevented the amplitude increase produced by BDNF. B, Average of the spike amplitude in the presence of inhibitors plus BDNF are displayed in the bars. C, PPR analysis of inhibitors used in A showed no differences. D, Time course of the population spike amplitude in the presence of the PLC‐γ inhibitor. This inhibitor alone increases the population spike compared to control and BDNF significantly reduced this effect on synaptic amplitude. E, Representative traces of population spikes in each condition. F, Averages of the spike amplitude in control, in the presence of U73122 and U73122 → BDNF is displayed in the bars (*F*_2,11_ = 17.224, *P* < 0.001, ANOVA with a post hoc Holm Sidak‐method). G, PPR analysis showed significant differences in the PPR of U73122 vs, control and U73122→BDNF (*F*_2,14_ = 10.396, *P* = 0.002, ANOVA with a post hoc Holm Sidak‐method). *P < 0.05 (n = 6; Mean ± SE)

Neurotrophins exert their effects through the activation of Trk receptors; these receptors, in turn, trigger several intracellular pathways. To understand the intracellular mechanisms that mediate the effects of BDNF and NT‐4/5, Trk receptors and the PI3K, MAPK and PLC‐γ signaling pathways were inhibited with K252a, LY294002, U0126, and U73122, respectively. Our data indicated that the inhibition of all of these factors impedes the modulatory effect of BDNF on corticostriatal transmission. Figure 2A and B summarizes the effects of the Trk inhibitor, the PI3K inhibitor LY294002 in the presence of BDNF, and the MAPK inhibitor in the presence of BDNF. The results show that both the PI3K and MAPK pathways are needed to produce the modulatory effects of BDNF on corticostriatal transmission. In the presence of the PLC inhibitor, BDNF reduced the spike amplitude, and these experiments are analyzed separately. The analysis of the paired‐pulse ratio (S2/S1) did not show changes among the control, LY294002, and LY294002 + BDNF groups. Similarly, the U0126 group did not show differences when compared to the control or BDNF + U0126 groups (Figure 2C).

Analyzing the role of PLC in BDNF, we found that U73122, a PLC inhibitor, increased the spike amplitude (Figure 2D). Surprisingly, BDNF reversed the effect of U73122 on the spike amplitude (Figure 2E‐F). PPR analysis suggested that the U73122action was presynaptically mediated (Figure 2G). The PLC inhibitor seems to affect calcium dynamics; therefore, in a series of experiments with low calcium concentration in the recording solution, the U73122‐dependent increase in the spike amplitude was eliminated, demonstrating that an extracellular calcium‐activated mechanism was responsible for this U73122 effect (data not shown).

### 3.4 Signaling pathways involved in the neuromodulation of NT‐4/5 in corticostriatal synapses

**Figure 3:**
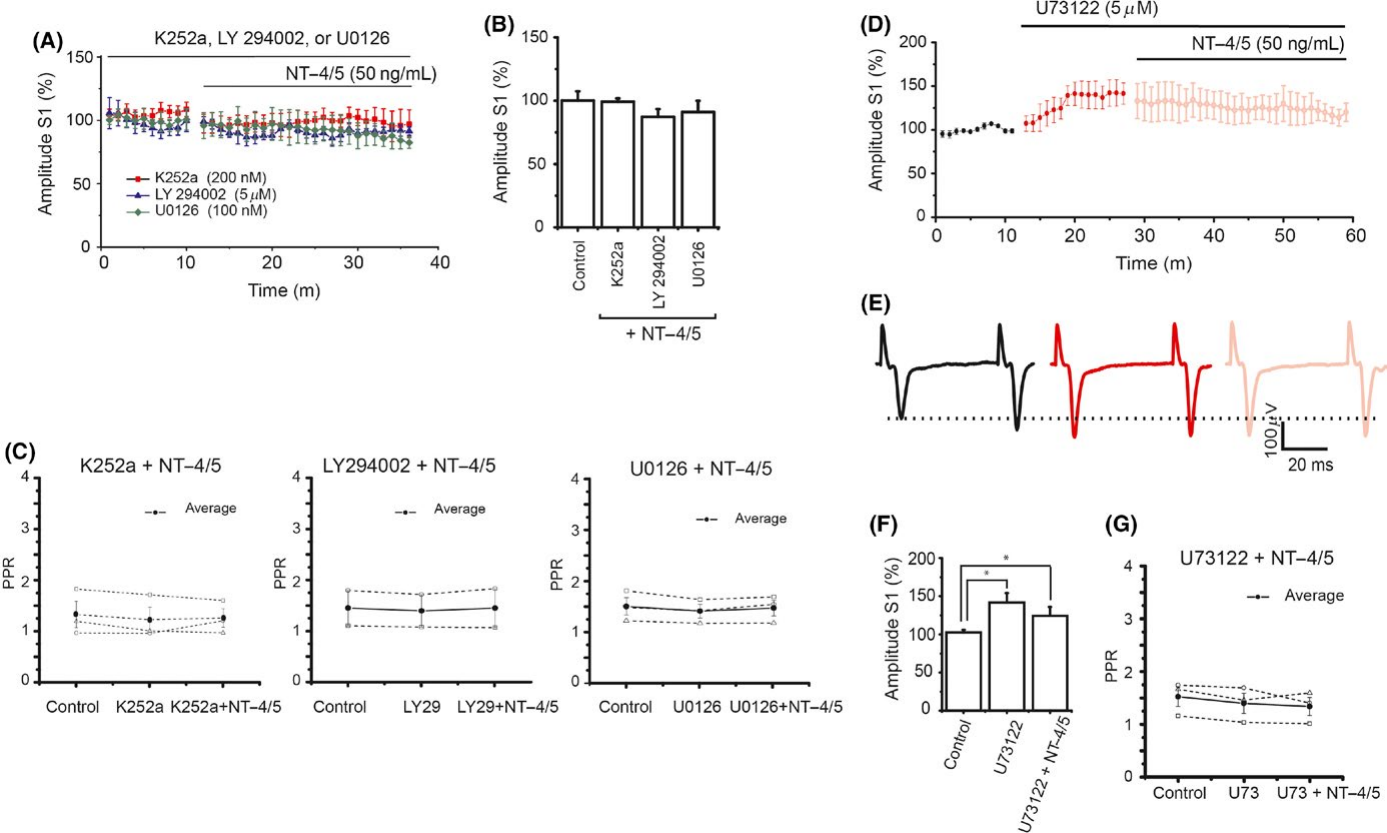
Signaling pathways associated with NT‐4/5 effects. A, Summary of the time course of the population spike amplitude in the presence of the inhibitors of Trk receptor, PI3K, and MAPK followed by NT‐4/5. All inhibitors prevented the amplitude increase produced by NT‐4/5. B, Amplitude averages of the NT‐4/5 in the presence of the inhibitors are displayed in the bars. C, Analysis of PPR did not show differences. D, Time course average of the spike amplitude experiments in the presence of the PLC‐γ inhibitor followed by NT‐4/5. Note that the spike increase induced by the PLC inhibitor did not change by NT‐4/5. E, Representative traces of population spikes from these experiments. F, Average of the effect of these experiments is displayed in the bars (*F*_3,29_ = 172.30, *P* < 0.001, ANOVA with a post hoc Holm Sidak‐method). G, PPR analysis did not show differences * *P* < 0.05 (n = 5; Mean ± SE)

In the evaluation of the signaling pathways involved in the NT‐4/5 modulation of corticostriatal synapses, blocking of the Trk receptors and the PI3K, or MAPK pathways prevented NT‐4/5 modulatory actions on corticostriatal synapses, similar to the effects seen with BDNF. Figure 3A‐C summarizes the effect of the Trk receptor inhibitor as well as the PI3K and MAPK signaling pathways. As described above the PLC inhibitor increases the synaptic spike amplitude, the further administration of NT‐4/5 did not modify the U73122 effect on spike amplitude in the corticostriatal synapse (Figure 3D‐F). PPR (S2/S1) analysis did not show a significant difference among the experimental conditions (Figure 3G).

### 3.5 NT‐4/5 antagonism of BDNF is independent from PLC activation

**Figure 4:**
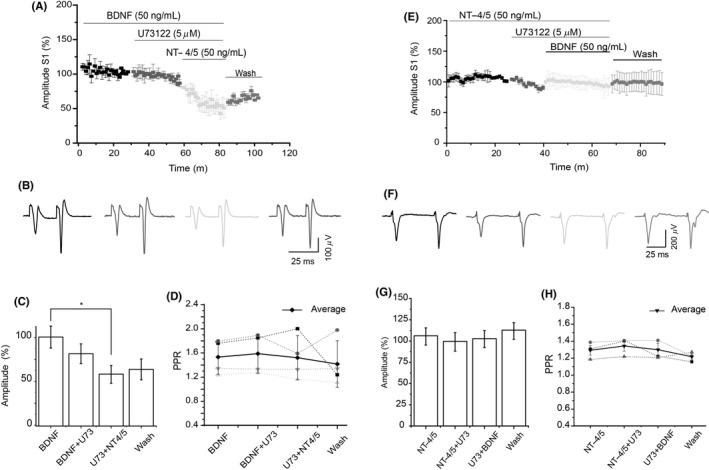
NT‐4/5 antagonism of BDNF is independent from PLC activation. A, Time course of the population spike amplitude in the presence of BDNF followed by PLC‐γ inhibitor and NT‐4/5. The PLC‐γ inhibitor did not have a significant effect on the population spike obtained with BDNF; however, the administration of NT‐4/5 reduced the population spike even in the presence of the PLC‐γ inhibitor. B, Representative traces of population spikes in each experimental of panel A. C, Amplitude average of the BDNF in the presence of the PLC inhibitor and NT‐4/5 are displayed in the bars. Differences were obtained between BDNF and BDNF + U73122 + NT‐4/5 (H_4_ = 18.846; *P* < 0.001, Kruskal‐Wallis ANOVA with a post hoc, Dunn's method). D, PPR did not change (n = 6, Mean ± SE). E, Time course of the population spike amplitude in the presence of NT‐4/5 followed by PLC‐γ, inhibitor, BDNF and wash. In the presence of NT‐4/5, the PLC‐γ inhibitor did not reduce significantly the population spike neither BDNF produced any further effect. F, Representative traces of population spikes from E. G, Amplitude average of theNT‐4/5 in the presence of the PLC inhibitor and BDNF are displayed in the bars. H, PPR in each experimental condition (n = 6, Mean ± SE)

Given that PLC‐γ activation by BDNF or NT‐4/5 is associated with glutamate release in cortical neurons^22^ and since the PLC inhibitor potentiates corticostriatal transmission, the following experiments investigated whether NT‐4/5 uses PLC signaling activation to antagonize the effect of BDNF. In experiments where U73122 followed BDNF administration, U73122 did not modify the BDNF effect. Moreover, the spike amplitude was significantly reduced when NT‐4/5 followed BDNF → U73122 incubation (Figure 4A‐C). These data showed that the antagonism exhibited by NT‐4/5 on the BDNF effect was not mediated by the activation of the PLC pathway. PPR analysis did not show differences among experimental groups (Figure 4D).

In another set of experiments, NT‐4/5 was administered first, followed by the PLC inhibitor; then, BDNF was applied. The PLC inhibitor did not modify the effect of NT‐4/5 (Figure 4E‐G). These data show that once NT‐4/5 triggered the signaling pathway, the PLC inhibitor did not affect synaptic transmission; furthermore, BDNF administration did not have any further effect on spike amplitude. PPR was not different amoung experimental conditions (Figure 4H).

### 3.6 BDNF and NT‐4/5 induced the phosphorylation of TrkB and PLC‐γ

**Figure 5:**
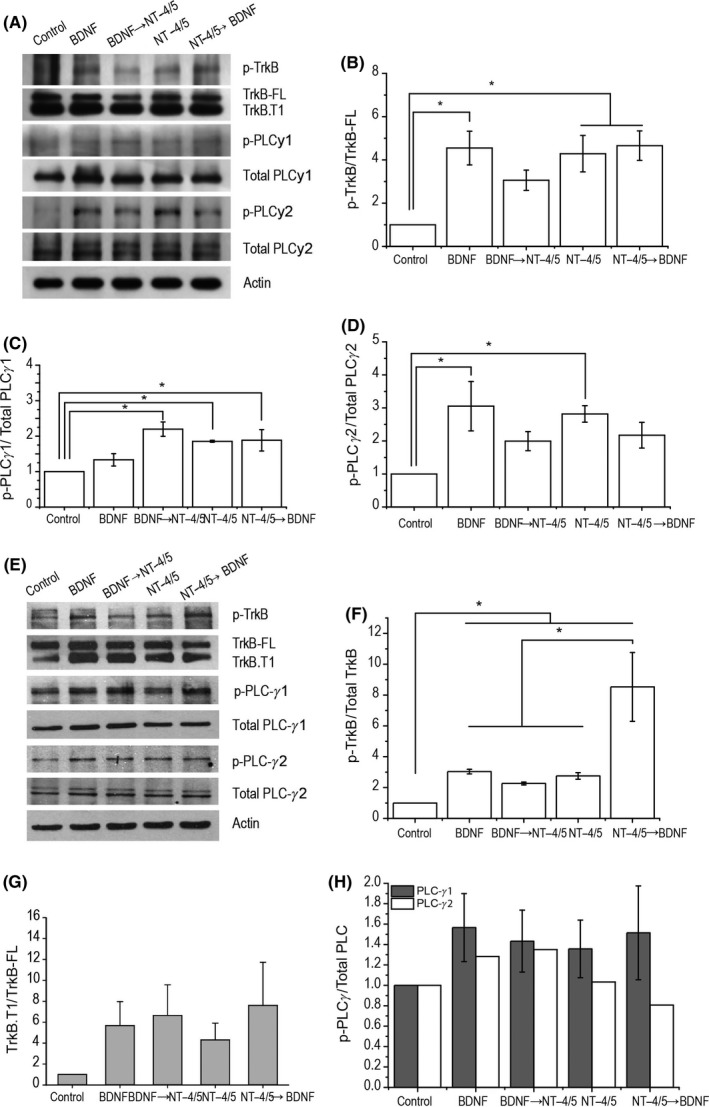
BDNF and NT‐4/5 activation of TrkB and PLC signaling, (Panel A‐D, 10´and Panel E‐H, 30´). A, Representative immunoblots images of tissue slices lysates at 10 m after neurotrophin treatment. B, Summary from the ratio of p‐TrkB/Total‐TrkB‐FL in each experimental condition is presented in the bars. Individual bands were normalized to control for each experimental group. Data are shown as Mean ± SE, (*P < 0.05; ANOVA with a post hoc Holm Sidak‐method). Neurotrophins significantly increased phosphorylation of TrkB, compared to control, but BDNF → NT‐4/5 phosphorylation was not different from control. C, PLCγ1 phosphorylation. p‐PLCγ1 ^Y783^ significantly increased in all treatments where NT‐4/5 was present, but not for BDNF alone. D, PLCγ2 phosphorylation. PLCγ2^Y759^ significantly increased only when BDNF or NT‐4/5 were present alone. E, Representative immunoblots of tissue slices lysates at 30 m after neurotrophin treatment. F, All neurotrophins increased p‐TrkB^Y816^, but phosphorylation induced by NT‐4/5 → BDNF, was significantly higher. G, Histogram shows the ratio of TrkB‐T/TrkB‐FL expression related to control. Note that TrkB.T isoform exhibited a nonsignificant increase. Each densitometry was normalized with actin as an endogenous control. H, The histogram represents the relative expression of p‐PLC‐γ1 Tyr/Total PLC‐γ1 and p‐PLC‐γ/total PLC‐γ2 ratios at 30 min, no differences were observed among groups.

TrkB activation by neurotrophin treatment was estimated by its phosphorylation state at 10 and 30 minutes. The presence of neurotrophins increased p‐TrkB^Y816^ in all treatments compared to that of the control, except for BDNF→NT‐4/5 when evaluated at 10 minutes (Figure 5A row 1, Figure 5B). These data suggested that NT‐4/5 inhibited p‐TrkB^Y816^. The evaluation at 30 minutes also showed an increase in p‐TrkB in all treatments, being higher under the NT‐4/5 → BDNF treatment (Figure 5E, row 1 and F). The analysis of the expression ratio of TrkB‐FL/TrkB.T1 showed no significant increase in all treatments compared to that of the control (Figure 5G).

The phosphorylation levels of the PLCγ isoforms p‐PLC‐γ1^Y783^ and p‐PLC‐γ2^Y759^ were investigated under BDNF, BDNF → NT‐4/5, NT4/5, or NT‐4/5 → BNDF exposure. At 10 minutes, the NT‐4/5, BDNF → NT‐4/5, and NT‐4/5 → BNDF treatments increased p‐PLC‐γ1 (Figure 5A, row 3, and C), while p‐PLCγ2 increased significantly in the presence of BDNF or NT‐4/5 compared to control expression; however, the phosphorylation levels did not change in the BDNF → NT‐4/5 or NT‐4/5 → BDNF treatments (Figure 5A, row 5, and D). The blots obtained for p‐PLC‐γ1^Tyr783^ and p‐PLC‐γ2^Y759^ at 30´ did not show differences among the treatments (Figure 5E, rows 3‐6, and H).

### 3.7 NT‐4/5 inhibits BDNF effect by downregulating TrkB‐FL and upregulating TrkB‐T.1

The accuracy of the biochemical analyses of receptors and signaling pathways in brain tissue homogenates is sometimes limited by the presence of mixed populations of neuronal and nonneuronal cells, which may exhibit basal levels of endogenous neurotrophin‐dependent phosphorylation or posttranslational modifications in proteins produced by active molecules. Cultured cell lines expressing desirable proteins in a more controlled environment have been frequently used to normalize the above‐mentioned concerns. Thus, to better analyze the participation of TrkB receptor isoforms with neurotrophin exposure, TrkB‐FL, TrkB.T1 or TrkB‐FL + TrkB.T1 were expressed independently in COS‐7 cells, and cell lysates were analyzed for PAGE and Western blotting. TrkB.T1 was selected because it regulates the TrkB‐FL receptor.

**Figure 6:**
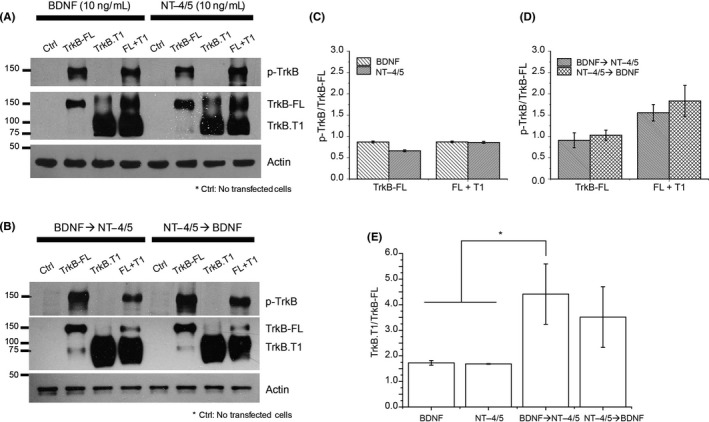
NT‐4/5 inhibits BDNF through upregulation of TrkB.T1 and downregulation of TrkB‐FL. A, Western blots of protein expression of TrkB receptors and its phosphorylation in COS7 cells. Primary antibodies dilution in PBS‐tw was p‐TrkB^Y816^ 1:1000 and α‐actin 1:1000 or TBS‐tw total‐TrkB 1:1000. BDNF and NT‐4/5 were used to evaluate the expression of p‐TrkB (145 KDa, 2nd, 4th, 6th, and 8th column), TrkB‐FL (145 KDa, 2nd, 4th, 6th, and 8th column) and TrkB‐T.1 (95 KDa, 3rd, 4th, 7th, 8th) under the BDNF and NT‐4/5 treatments. B, Western blots of protein expression of TrkB receptors isoforms under, BDNF → NT‐4/5 and NT‐4/5 → BDNF treatments. Primary antibodies dilution was 1:10 000. BDNF → NT‐4/5 induced, p‐TrkB of TrkB‐FL (145 KDa, 2nd column), upregulation of TrkB.T1 (95 KDa, 3rd and 4th columns), and downregulation of TrkB‐FL, in addition to reduction in p‐TrkB when both isoforms were coexpressed in COS‐7 cells (4th column). NT‐4/5 → BDNF induced p‐TrkB and TrkB‐FL (6th column), TrkB.T1 (7th column) expression. When the two isoforms were coexpressed in COS 7 cells, TrkB‐FL expression was reduced (8th vs to 6th column), without affecting p‐TrkB and TrkB.T1 (8th column). Control = nontransfected COS‐7 cells. C, Summary of the p‐TrkB/Total‐TrkB‐FL in panel A is presented in the bars. There were no differences in the p‐TrkB level expression to BDNF or NT‐4/5 exposure. D, Summary of the p‐TrkB/Total‐TrkB‐FL from panel B is presented in the bars. There were no differences in the TrkB‐FL level expression, but phosphorylation changed when FL and T.1 isoforms were coexpressed. E, Analysis of optical density of TrkB.T1/TrkB‐FL bands ratio increased for BDNF → NT‐4/5 and NT‐4/5 → BDNF treatments (panel B), compared to BDNF and NT‐4/5 (panel A; *F*_3,7_ = 62.007, *P* < 0.001, ANOVA with a post hoc Holm Sidak‐method). *P < 0.05

Brain‐derived neurotrophic factor (BDNF) and NT‐4/5 treatments strengthen the expression of p‐TrkB, TrkB‐FL, TrkB.T1, and TrkB‐FL+T1 receptors compared to untreated cells. No basal expression of any receptor was obtained in control nontransfected cells, and, no expression of p‐TrkB was found in cells expressing TrkB.T1, which is the truncated isoform that lacks tyrosine kinase activity (Figure 6A).

The BDNF → NT‐4/5 treatment induced the occurrence of p‐TrkB and the normal expression of TrkB‐FL but upregulated TrkB.T1 expression compared to BDNF treatment alone (Figure 6A 3rd column vs Figure 6B, 3rd column). When receptors were coexpressed in COS‐7 cells, TrkB.T1 was expressed, and p‐TrkB levels decreased; however, TrkB‐FL was downregulated (Figure 6B). The NT‐4/5 → BDNF treatment induced similar results to the BDNF → NT‐4/5 treatment (Figure 6B). Nevertheless, the upregulation of TrkB.T1 produced was minor (Figure 6B). When both receptor isoforms were coexpressed in COS‐7 cells, the NT‐4/5 → BDNF treatment produced a robust activation of p‐TrkB but a slight expression of TrkB‐FL while TrkB.T1 expression level was similar to that obtained for NT‐4/5 (panel A). These results confirmed that the NT‐4/5 antagonism of BDNF activity is regulated via the upregulation of the TrkB.T1 receptor isoform, which, in turn, downregulates TrkB‐FL activation.

## 4 DISCUSSION

The main finding in our study was the discovery that NT‐4/5 inhibits BDNF activity by stimulating the TrkB.T1 isoform and downregulating the TrkB‐FL receptor. Previously, we showed that BDNF and NT‐4/5 potentiate corticostriatal transmission to different degrees.^21^ Since both neurotrophins are present in the striatum and both target striatal medium spiny neurons, we questioned whether their effects were additive, no additive, or occlusive. In particular, we evaluated the physiological relevance of the sequential administration of BDNF → NT‐4/5 and NT‐4/5 → BDNF. The data obtained demonstrated that if BDNF was administered first, its effects were antagonized by the subsequent administration of NT‐4/5. However, the NT4/5 modulation of corticostriatal synapses was unaltered upon BDNF application.

To understand the mechanisms underlying our results, we explored signaling pathways and receptor involvement. The inhibition of the TrkB, PI3K, and MAPK signaling pathways impeded BDNF or NT‐4/5 modulation on corticostriatal synapses, demonstrating that these signaling pathways are essential for glutamatergic transmission modulation by neurotrophins, as seen in other cerebral areas.^14^, ^16^, ^17^, ^23^, ^24^, ^25^, ^26^, ^27^, ^28^, ^29^, ^30^

The U73122 increased the spike amplitude by itself compared to the control. This result was surprising since the U73122 is a specific inhibitor of PLC‐γ. Nonetheless, several studies have suggested that U73122 has unrelated effects on the inhibition of PLC, including the depletion of Ca^2+^ intracellular stores,^31^, ^32^ which explains why the decrease in extracellular calcium prevented the effects of U73122 on spike amplitude. U73122 also displayed different results depending on the neurotrophin application that followed its administration, which demonstrated that BDNF and NT‐4/5 do not exert their effects via the same mechanism when they coexist.

The experiments in which the treatment sequence was BDNF → PLC inhibitor → NT‐4/5, NT‐4/5 kept antagonizing BDNF effect, indicating that NT‐4/5 inhibition of BDNF was independent from PLC‐γ activation. Nonetheless, in the NT‐4/5 → PLC inhibitor → BDNF treatment, BDNF did not decrease the spikes amplitude as it did in the experiments preincubated only with the PLC blocker. It is possible that U73122 changed the neurotrophic effect by modifying calcium influx.

### 4.1 NT‐4/5 inhibits BDNF effect through the activation of the TrkB.T1 isoform

We speculated that BDNF and NT‐4/5 had differential interactions with the TrkB receptor isoforms in the electrophysiology experiments. Thus, the phosphorylation of p‐TrkB^Y816^ was estimated for each neurotrophin 10 or 30 minutes after treatment. BDNF and NT‐4/5 independently induced similar p‐TrkB levels; however, when the treatment order was BDNF → NT‐4/5, p‐TrkB was reduced. If the treatment order was NT4/5 → BDNF, p‐TrkB increased. These results suggested that NT‐4/5 antagonism resulted in the interaction of NT‐4/5 with one of the TrkB receptor isoforms after BDNF has phosphorylated TrkB receptor. TrkB‐FL and TrkB.T1 are present in striatal neurons,^33^ the TrkB.T1 isoform is a natural antagonist that negatively modulates TrkB‐FL activity.^34^, ^35^, ^36^ We hypothesized that NT‐4/5, by interacting with TrkB.T1, inhibited TrkB‐FL. To evaluate this hypothesis, the TrkB‐FL, TrkB.T1, and TrkB‐FL + TrkB‐T1 receptor isoforms were independently expressed in COS‐7 cells, and their expression was evaluated under BDNF, NT‐4/5, BDNF → NT‐4/5, or NT‐4/5 → BDNF treatment. Both BDNF and NT‐4/5 induced the expression of the 2 isoforms, but the BDNF → NT‐4/5 treatment resulted in the downregulation of TrkB‐FL and p‐TrkB when both TrkB‐FL and TrkB.T1 receptor isoforms were coexpressed in COS‐7 cells. The NT‐4/5 → BDNF treatment also induced robust expression of TrkB‐FL and TrkB.T1 but did not modify the p‐TrkB level. Hence, these results confirmed that the interaction of NT‐4/5 and TrkB.T1 downregulated TrkB‐FL, reduced p‐TrkB, and, consequently, reversed the effect of BDNF. Panel B from Figure 6 displayed a 75 kDa band, which may correspond to the truncated receptor TrkB‐T‐TK that lacks the C‐terminal of the TrkB‐FL, but retains the tyrosine kinase activity.^37^ It is unknown whether this other truncated receptor isoform participates in the NT‐4/5 regulation of BDNF activity.

Although occlusive effects among neurotrophins have been described previously in the nervous system,^38^, ^39^, ^40^ the inhibitory effect of NT‐4/5 on BDNF has not been reported in the striatum. Despite the work describing that BDNF and NT4/5 produce different patterns of dendritic growth,^41^ most of the studies indicate that both neurotrophins exhibit similar effects.^29^

Our results in COS‐7 cells indicate that NT‐4/5 upregulates TrkB.T1 and downregulates TrkB‐FL. TrkB.T1 was evaluated in COS‐7 cells because it is the predominant isoform in the adult brain^42^, ^43^; furthermore, it regulates TrkB‐FL function,^44^ and its main role is to inhibit TrkB‐FL signaling by forming inactive heterodimers with the TrkB‐FL receptor^36^ or by sequestering neurotrophins and preventing their binding to TrkB‐FL.^34^, ^35^ We do not disregard the participation of the low‐affinity p75 receptor in NT‐4/5 antagonism.

Finally, our study shows that BDNF and NT‐4/5 stimulate TrkB receptors differently under certain physiological conditions. There is evidence of a different interaction between BDNF and NT‐4/5 on the TrkB receptor; for example, the substitution of cystein 354 for serine 354 on the second immunoglobulin motif of the extracellular domain prevents the morphological transformation of NIH3T3 cells in the presence of NT‐4/5 but not BDNF.^45^ Furthermore, the mutation of the Shc adaptor protein impedes sensory neuron survival in the presence of NT‐4/5 but not BDNF.^46^ BDNF replacement by knock‐in of the NT‐4/5 gene into the BDNF locus of mice showed that only those with NT‐4/5 substitution were viable, demonstrating distinct activities for both neurotrophins.^47^ Finally, endocytic trafficking of TrkB receptors is differentially modulated by NT‐4/5 and BDNF.^48^

What is the physiological meaning of the antagonistic effect of NT‐4/5 on BDNF in the striatum? Perhaps, differential modulation is necessary to reduce the excess excitation produced by the increase in synaptic spikes in the presence of BDNF and to maintain synaptic activity at basal levels. In terms of the circuitry, cortical influences first activate interneurons and medium spiny neurons later^49^; if interneurons or axonal collaterals release NT‐4/5, corticostriatal transmission would be increased. However, if the corticostriatal pathway releases BDNF first, the subsequent activation of interneurons or axonal collaterals of MSNs would release NT‐4/5 and might reduce the synaptic augmentation produced by BDNF. Further experiments are needed to test this hypothesis.

## CONFLICT OF INTEREST

The authors declare no conflict of interest.
